# Successful Management of Advanced Olfactory Esthesioneuroblastoma: A Case Report

**DOI:** 10.1002/ccr3.71208

**Published:** 2025-10-15

**Authors:** Chiraz Halwani, Sana Ferchichi, Mariem Messelmani

**Affiliations:** ^1^ Faculty of Medicine of Tunis University of Tunis el Manar Tunis Tunisia; ^2^ Department of Otorhinolaryngology Military Hospital of Tunis Tunis Tunisia; ^3^ Department of Otorhinolaryngology Mohamed Taher Maamouri University Hospital Nabeul Tunisia; ^4^ Department of Neurology Military Hospital of Tunis Tunis Tunisia

**Keywords:** differential diagnosis, esthesioneuroblastoma, management, surgery

## Abstract

Early imaging and biopsy are crucial for diagnosing esthesioneuroblastoma. Multimodal treatment—endoscopic or combined surgery, adjuvant radiotherapy, and selective chemotherapy—offers the best outcomes. Long‐term follow‐up is essential to detect recurrences and manage complications like optic neuritis. Multidisciplinary coordination optimizes care in this anatomically complex, recurrent‐prone tumor.

## 1 Introduction

Management of esthesioneuroblastoma (ENB) is not straightforward and presents several challenges due to the tumor's rarity, complex anatomical location, and variable biological behavior. While advancements in treatment modalities have improved outcomes, ENB remains a malignancy with a high probability of locoregional recurrence and the need for individualized interventions. Its rarity contributes to the absence of a unified staging system and treatment protocol [1]. The optimal treatment regimen remains unclear, making it difficult to establish a standard approach [2].

This case report aims to highlight the clinical presentation, diagnostic challenges, and treatment strategies for esthesioneuroblastoma, with a focus on the role of multimodal management and long‐term follow‐up in preventing recurrence and managing complications.

## 2 Case History/Examination

A 53‐year‐old patient with no significant medical history presented with visual blurring, exophthalmos, and a progressive swelling of the medial canthus of the left eye over one month.

**FIGURE 1:**
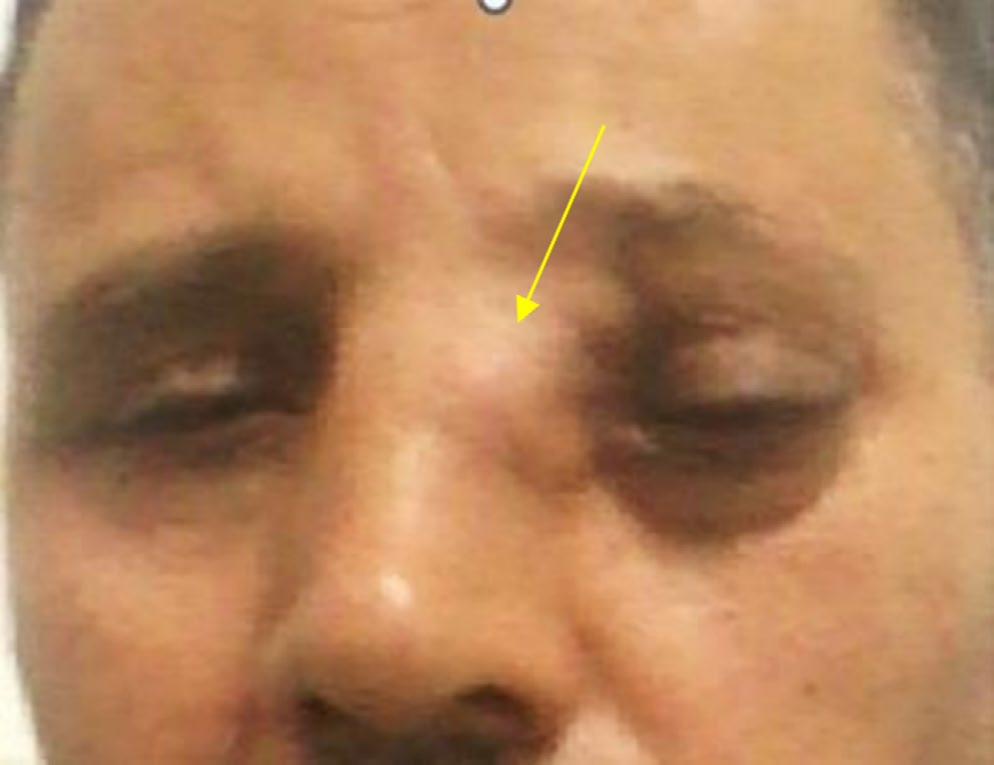
Nasal root widening with obliteration of the medial canthus of the left eye and inferior‐lateral displacement of the globe.

Physical examination revealed nasal root widening with obliteration of the medial canthus of the left eye and inferior‐lateral displacement of the globe (Figure 1). Nasal endoscopy identified a fleshy, tumor‐like mass arising from the left superior meatus, displacing the nasal septum contralaterally without obstructing nasal airflow. The remainder of the examination was unremarkable, with a clear nasopharynx and no cervical lymphadenopathy.

## 3 Differential Diagnosis, Investigations and Treatment

**FIGURE 2:**
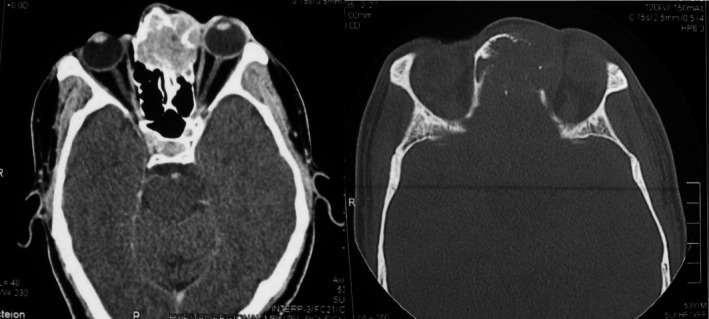
CT scan of the facial mass in axial section ethmoido‐frontal tissue process with heterogeneous enhancement following contrast injection, showing bone lysis of the inner wall of the left orbit.

Facial CT scan showed a suspicious ethmoidal‐frontal soft tissue mass, associated with fronto‐orbital bone erosion and extension into the intracranial and left orbital compartments (Figure 2).

**FIGURE 3:**
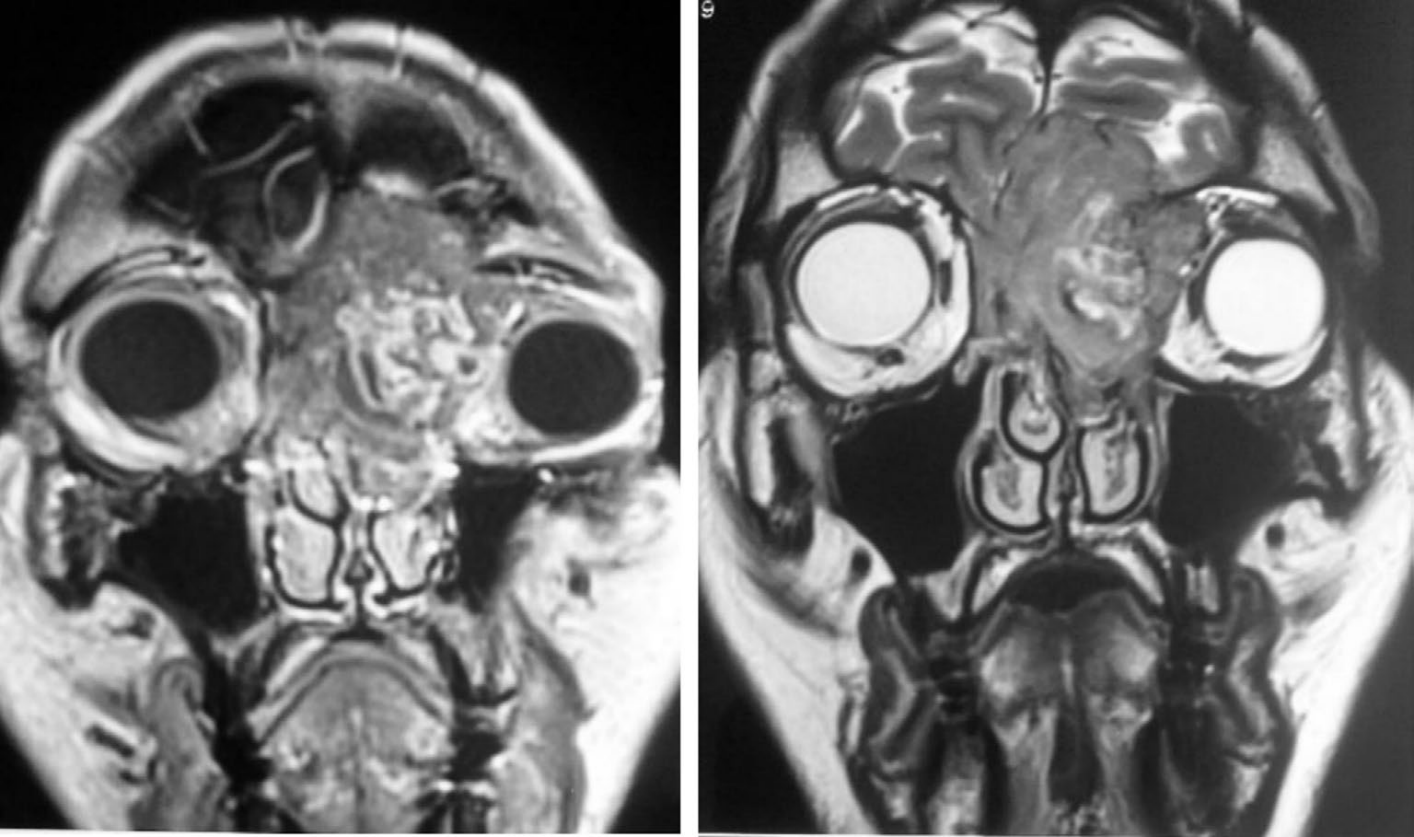
Facial mass MRI in coronal section with T1 and T2 sequences: Tissue process is iso‐intense on T1, showing heterogeneous enhancement with iso‐ and hyper‐intensity on T2, with orbital, intracranial, and intraorbital extension.

On MRI, the lesion exhibited iso‐intensity on T1‐ and T2‐weighted sequences with heterogeneous contrast enhancement. The tumor was centered on the left ethmoidal air cells, infiltrating the frontal bone with basi‐frontal intracranial extension while remaining extra‐axial. The lesion extended intra‐orbitally on the left side, with erosion of the lamina papyracea and invasion of both the extraconal and intraconal spaces (Figure 3). The ADC value was 0.9 × 10^−3^ mm^2^/s.

At that point, we considered several possible diagnoses based on the tumor's location and appearance. Esthesioneuroblastoma was high on the list, given its tendency to arise in the upper nasal cavity. We also thought about sinonasal undifferentiated carcinoma (SNUC), which is known to grow quickly and behave aggressively, as well as lymphoma, since it can show up as a soft mass without much early bone damage. Other possibilities included adenoid cystic carcinoma and squamous cell carcinoma. We also briefly considered vascular tumors like hemangiopericytoma, though they seemed less likely. Benign conditions like mucoceles or osteomas were low on the list, mainly because the symptoms had progressed quickly and caused noticeable displacement of the eye.

**FIGURE 4:**
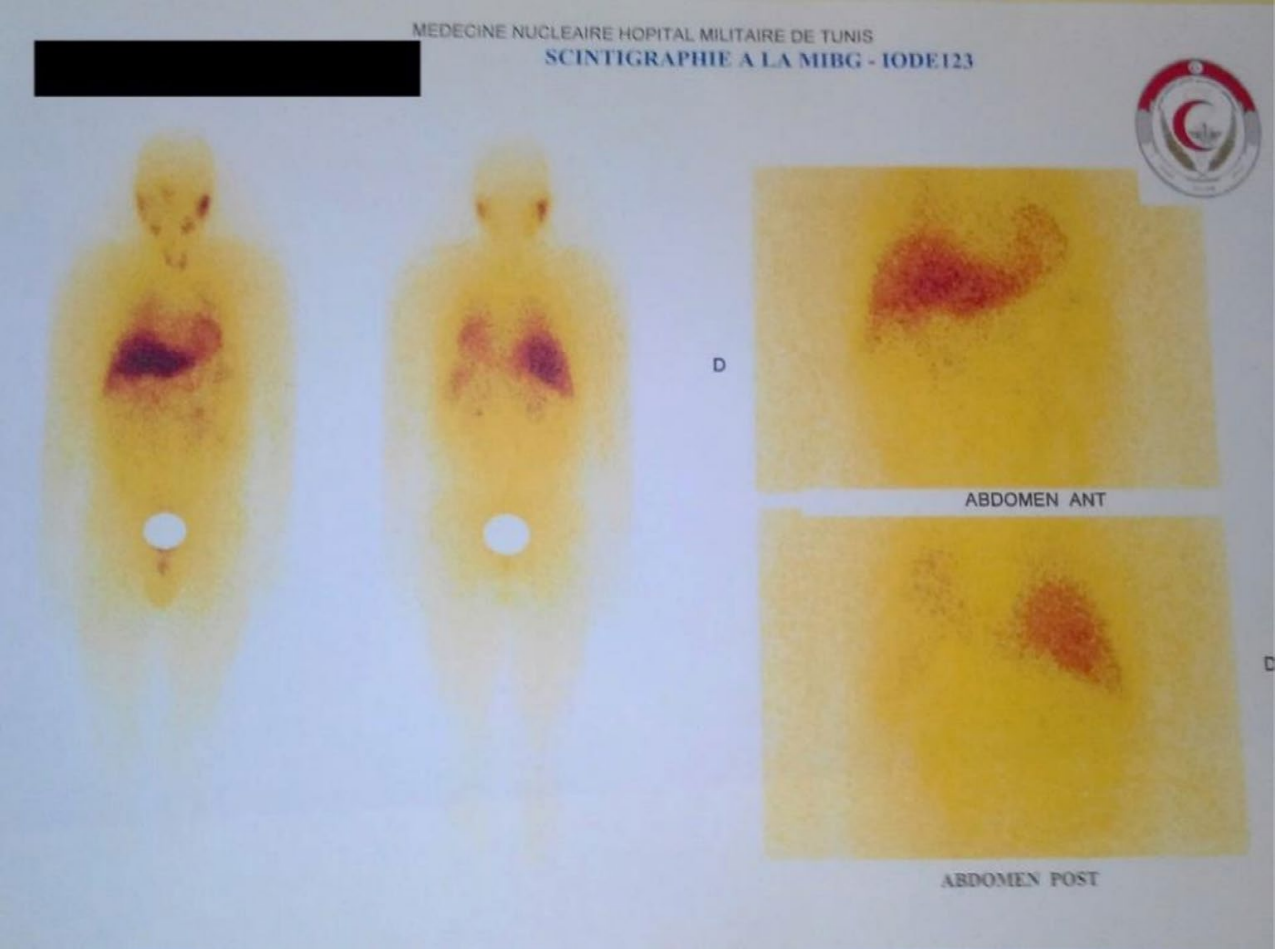
MIBG scintigraphy: No suspicious uptake, with physiological tracer visualization in the salivary glands, heart, and liver.

A systemic assessment was performed to rule out metastatic disease: MIBG scintigraphy did not reveal any uptake suggestive of a primary or secondary neuroendocrine tumor. Similarly, the thoraco‐abdominopelvic CT scan showed no signs of distant metastases (Figure 4).

Histopathological analysis of the biopsy specimen confirmed the diagnosis of an olfactory esthesioneuroblastoma.

Concomitant radio‐chemotherapy was initiated: Chemotherapy consisted of three cycles of VP16‐CDDP (Etoposide‐Cisplatin), administered on Days 1 and 21, and radiotherapy was delivered with a total dose of 45 Gy using a conventional fractionation schedule.

Post‐treatment MRI demonstrated tumor stabilization, paving the way for surgical intervention via a combined otolaryngologic and neurosurgical approach.

The patient underwent a two‐step surgical resection. The first stage consisted of an exclusive endoscopic approach. Due to residual disease, a secondary bicoronal approach was performed, achieving complete tumor resection with skull base reconstruction to repair the osseous defect.

## 4 Conclusion and Results (Outcome and Follow‐Up)

The postoperative period was complicated by multiple episodes of local superinfection, requiring targeted antimicrobial therapy.

Longitudinal follow‐up, conducted via endoscopic examination and serial imaging since 2017, revealed the development of post‐radiation optic neuritis, which led to progressive and ultimately irreversible vision loss in the affected eye. Close clinical and radiological surveillance remains ongoing to monitor for tumor recurrence and delayed complications.

## 5 Discussion

ENB originates from the olfactory neuroepithelium in the nasal cavity, often extending to the anterior cranial base [3, 4]. This location poses challenges for complete surgical resection and increases the risk of intracranial extension [1, 5]. ENB exhibits a broad spectrum of biological behavior, making it difficult to predict its metastatic tendencies and response to various therapies [5].

The Kadish staging system is commonly used, but other systems like the Dulguerov modification of the TNM classification are also employed [6, 7]. The majority of tumors (84.4%) as in our case, were within Kadish C stage, 79.7% were within T3 or T4, and 64.0% were within Hyams grade III or IV [1].

Due to the complexities of ENB, a multidisciplinary approach involving surgery, radiation therapy, and chemotherapy is often necessary [6, 8].

Surgical resection is a primary treatment modality for ENB, aiming for complete en bloc resection of the tumor [8, 9]. Endoscopic resection, including endoscopically assisted approaches, has shown comparable survival and recurrence rates with decreased patient morbidity [1, 9]. Endoscopic surgery resulted in significantly better 5‐year progression‐free survival (PFS) compared to open surgery (61.7% vs. 22.2%, *p* < 0.001) [1].

Open craniofacial resection has been the traditional approach, allowing for wide resection margins and control of the tumor [8, 9]. Achieving negative margins during surgery is crucial for local control and survival [8].

Adjuvant radiation therapy (RT) is frequently combined with surgery to improve local control and overall survival [1, 2]. Surgery combined with radiotherapy, with or without chemotherapy, results in significantly better overall survival compared to surgery alone and radiotherapy alone (*p* = 0.0064) [1].

Intensity‐modulated radiotherapy (IMRT) and carbon ion radiotherapy (CIRT) are advanced radiation techniques that might improve local tumor control [4, 10]. Re‐irradiation with CIRT seems to be a feasible and effective treatment method in ENB [4].

The role of chemotherapy in ENB management is less defined, and its use is often reserved for advanced or recurrent cases [5, 11].

Preoperative chemotherapy may be used to reduce tumor burden and improve the feasibility of surgical resection [8]. Postoperative chemotherapy may be administered to target residual disease and prevent recurrence [8]. Various chemotherapy regimens, such as vincristine, ifosfamide, doxorubicin, and etoposide, have been used, but their effectiveness varies [12].

Several factors can influence the prognosis of ENB, including:

Intracranial Extension [1], cervical Lymph Node Metastasis [1, 10], High Hyams grade (IV) [1, 6], advanced Kadish stage [8], advanced age [8], skin Involvements [2].

Reported survival rates for ENB vary depending on the stage, treatment approach, and other prognostic factors [1, 2, 7].

Surgery combined with radiotherapy resulted in significantly better overall survival compared to surgery alone and radiotherapy alone [1]. In one study, the 5‐year overall survival rate was 42.7%, and the 10‐year rate was 28.9% [2]. Another study reported a 5‐year overall survival of 84.4% for patients receiving surgery combined with radiotherapy [1]. A study analyzing 97 patients reported an overall crude survival of 65.4% and a determinate survival (corrected for intercurrent disease) of 70.8% [7].

Advanced radiation techniques, such as IMRT and CIRT, have shown promising results in local tumor control and survival [4]. IMRT is a radiation technique that allows for the delivery of highly conformal radiation doses to the tumor while minimizing exposure to surrounding healthy tissues [10].

Cihang Bao et al. evaluated the efficacy and safety of IMRT in 52 ENB patients and reported acceptable 3‐year outcomes in terms of overall survival (89.7%), local progression‐free survival (89.7%), and regional progression‐free survival (95.1%) rates without substantial late adverse effects [10]. No severe (grade 3 or 4) IMRT‐induced acute toxicity was observed in the study, and severe late toxicities were infrequent (11.5%), including dysosmia (3.8%), hearing loss (3.8%), radiation brain injury (1.9%), and temporal lobe necrosis (1.9%) [10]. Late ocular toxicity secondary to IMRT was not observed [10].

CIRT is another advanced radiation technique that uses carbon ions to deliver radiation doses to the tumor [4].

Given that ENB arises from the olfactory neuroepithelium, preserving the sense of smell is an important consideration in treatment planning [9]. Tajudeen et al. reported a multi‐institutional series assessing smell outcomes of patients who underwent unilateral endoscopic resection of esthesioneuroblastoma with preservation of the contralateral olfactory bulb [9]. This approach may offer the possibility of preserving olfactory function while achieving adequate tumor control [9].

Despite advancements in diagnostic studies and treatment approaches, some data suggest that ENB survival has remained unchanged over the years [13]. Vuong et al. accessed the Surveillance, Epidemiology, and End Results (SEER) program to identify ENB cases from 1998 to 2016 and found that there has been no change in survival rates for patients with ENB over the past two decades (*p* = 0.793) [13].

The study by Vuong et al. confirmed that surgical resection and adjuvant radiotherapy are associated with improved patient survival, whereas the use of chemotherapy should be considered carefully [13]. Due to the potential for local and distant recurrence, long‐term follow‐up is essential for patients with ENB [5, 6].

## 6 Conclusion

Management of esthesioneuroblastoma is complex and requires a multidisciplinary approach. While surgery and radiation therapy are the mainstays of treatment, the role of chemotherapy is less defined. Despite advancements in treatment modalities, ENB survival has not significantly improved over the past two decades, highlighting the need for improved efforts to develop appropriate individualized interventions for this rare tumor entity.

## Author Contributions

**Chiraz Halwani:** conceptualization, data curation, writing – original draft, writing – review and editing. **Sana Ferchichi:** conceptualization, data curation, resources, writing – original draft. **Mariem Messelmani:** investigation, validation, visualization.

## Ethics Statement

The authors have nothing to report.

## Consent

Written Informed Consent from the patient for the publication of this case report is available from the corresponding author upon reasonable request.

## Conflicts of Interest

The authors declare no conflicts of interest.

## Data Availability

The data supporting the findings of this study is available from the corresponding author upon reasonable request.

## References

[1] Q. Zeng , Y. Tian , Y. He , et al., “Long‐Term Survival Outcomes and Treatment Experience of 64 Patients With Esthesioneuroblastoma,” Frontiers in Oncology 11 (2021): 624960, 10.3389/fonc.2021.624960. 33747939 PMC7969639

[2] Y. Yuan , J. Ye , H. Qiu , et al., “Exploration of the Optimal Treatment Regimes for Esthesioneuroblastoma: A Single Center Experience in China,” Journal of Cancer 9, no. 4 (2018): 777–782, 10.7150/jca.21605. PMC5743725 29290783

[3] T. Ow , E. Hanna , D. Roberts , et al., “Optimization of Long‐Term Outcomes for Patients With Esthesioneuroblastoma,” Head & Neck 36, no. 4 (2014): 524–529, 10.1002/hed.23327. 23780581

[4] J. Liermann , M. Syed , T. Held , et al., “Advanced Radiation Techniques in the Treatment of Esthesioneuroblastoma: A 7‐Year Single‐Institution Clinical Experience,” Cancers 10, no. 11 (2018): 457, 10.3390/cancers10110457. 30463343 PMC6267306

[5] R. Rodas , B. Erkman‐Balis , and D. Cahill , “Late Intracranial Metastasis From Esthesioneuroblastoma: Case Report and Review of the Literature,” Neurosurgery 19, no. 4 (1986): 610–613, 10.1227/00006123-198610000-00020. 3785601

[6] Z. Alami , F. Farhane , A. Bouziane , et al., “Management of Esthesioneuroblastoma: A Retrospective Study of 6 Cases and Literature Review,” Case Reports in Oncology 15, no. 1 (2022): 312–318, 10.1159/000521736. 35431860 PMC8958627

[7] D. Elkon , S. Hightower , M. Lim , R. Cantrell , and W. Constable , “Esthesioneuroblastoma,” Cancer 44, no. 3 (1979): 1087–1094, 10.1002/1097-0142(197909)44:3<1087::AID-CNCR2820440343>3.0.CO;2-A. 383268

[8] R. Polin , J. Sheehan , A. G. Chenelle , et al., “The Role of Preoperative Adjuvant Treatment in the Management of Esthesioneuroblastoma: The University of Virginia Experience,” Neurosurgery 42, no. 5 (1998): 1029–1033, 10.1097/00006123-199805000-00045. 9588547

[9] B. Tajudeen , N. Adappa , E. Kuan , et al., “Smell Preservation Following Endoscopic Unilateral Resection of Esthesioneuroblastoma: A Multi‐Institutional Experience,” International Forum of Allergy & Rhinology 6, no. 5 (2016): 493–496, 10.1002/alr.21794. 27431053

[10] C. Bao , W. Hu , J. Hu , Y. Dong , J. J. Lu , and L. Kong , “Intensity‐Modulated Radiation Therapy for Esthesioneuroblastoma: 10‐Year Experience of a Single Institute,” Frontiers in Oncology 10 (2020): 1158, 10.3389/fonc.2020.01158. 32766154 PMC7379860

[11] L. Cranmer and B. Chau , “Chemotherapy in the Management of Olfactory Neuroblastoma/Esthesioneuroblastoma: An Analysis of the SEER 1973–2015 Database,” Journal of Clinical Oncology 37, no. 15_suppl (2019): e17573, 10.1200/JCO.2019.37.15_suppl.e17573.

[12] E. B. Dinca , M. Radatz , J. Rowe , and A. Kemeny , “ *gamma* Knife Radiosurgery for Recurrent Intracranial Olfactory Neuroblastoma (Esthesioneuroblastoma): A Case Report,” Journal of Medical Case Reports 6 (2012): 240, 10.1186/1752-1947-6-240. 22889266 PMC3459740

[13] H. Vuong , D. D. Nguyen , E. El‐Rassi , T. Ngo , and I. Dunn , “Absence of Survival Improvement for Patients With Esthesioneuroblastoma Over the Past Two Decades: A Population‐Based Study,” Preprint (2021), 10.21203/RS.3.RS-600525/v1. 34628034

